# Left Atrial Appendage Occlusion: Expanding Indications and New Developments

**DOI:** 10.1016/j.shj.2024.100354

**Published:** 2024-08-03

**Authors:** Grant W. Reed, Shady Nakhla, Rhonda Miyasaka, Serge Harb, Mohamed Kanj, Ousamma Wazni, Samir R. Kapadia, Amar Krishnaswamy

**Affiliations:** Department of Cardiovascular Medicine, Cleveland Clinic, Cleveland, Ohio

**Keywords:** Atrial fibrillation, Anticoagulation, Left atrial appendage occlusion

## Abstract

Percutaneous left atrial appendage occlusion (LAAO) is recommended in several major international society guidelines as a viable alternative to therapeutic anticoagulation for the prevention of ischemic stroke in patients with nonvalvular atrial fibrillation or flutter. Recent innovations in device development have improved the safety and procedural success of LAAO, further fueling enthusiasm for expanding its indications beyond patients with high-bleeding risk from oral anticoagulation use. It is the aim of this review to provide historical context in addition to recent updates and upcoming developments and provide practical suggestions on how best to care for patients who are candidates for LAAO in contemporary practice. Recent data comparing the safety and efficacy of post-LAAO antiplatelet vs. antithrombotic therapy will be highlighted, with specific recommendations regarding which patients are best suited for each strategy. We will also address the safety and practical considerations provided by emerging trials on concomitant LAAO during other structural heart interventions such as transcatheter aortic valve replacement and mitral valve interventions, as well as electrophysiology procedures including catheter ablation for atrial fibrillation and pacemaker implantation. Practical considerations for the use of transesophageal echocardiography or intracardiac echocardiography for procedural guidance will also be discussed. As the evidence supporting LAAO continues to evolve, this review will serve as a primer on the recent and upcoming advances in device technology and management strategies positioned to further push LAAO forward into the future.

## Highlights

• Left atrial appendage occlusion (LAAO) is safe and effective in reducing stroke patients with atrial fibrillation.
• New devices and techniques have dramatically improved the safety and efficacy of LAAO.
• LAAO is emerging as an alternative to oral anticoagulation in many patients with atrial fibrillation.
• Combined LAAO ​+ ​structural/electrophysiology procedures may further improve safety and cost.

## Introduction

In the pursuit of more effective stroke prevention strategies, percutaneous left atrial appendage occlusion (LAAO) has emerged as a compelling therapeutic strategy in patients with nonvalvular atrial fibrillation or flutter (NVAF) who are at increased risk of thromboembolic events and bleeding complications with the use of oral anticoagulants (OACs).^1^, ^2^, ^3^, ^4^ While OACs have been the cornerstone for ischemic stroke prevention in NVAF for decades, adherence to OACs can be problematic due to bleeding complications, medication side effects, and cost, among other reasons.^5^ Certain studies indicate only approximately 50% of patients with an indication for OAC due to NVAF actually take an OAC, and among those that do, adherence is poor over long-term follow-up even with direct oral anticoagulants (DOACs).^6^^,^^7^ Given this, and with the knowledge that more than 90% of atrial thrombi responsible for ischemic stroke in NVAF originate within the left atrial appendage (LAA),^3^ LAAO has found its role as an appealing alternative to OAC.

While early studies of LAAO focused on patients with a higher risk of stroke and bleeding complications,^8^^,^^9^ progressive innovations in device technology, refinement of procedural techniques, and simplification of antithrombotic strategies post-LAAO have broadened the applicability of LAAO.^3^ The most recent generation of LAAO devices allows for implantation across a wider range of LAA anatomies with shorter procedural times and ease of implantation. They boast enhanced procedural safety, improved closure success rates, better outcomes, and improved patient satisfaction.^10^, ^11^, ^12^, ^13^ Data that supports the risk/benefit ratio of LAAO has improved and is more favorable when evaluated over a longer time horizon, as the long-term complications of OAC use are additive and can be significant for up to 5 years.^14^

Accordingly, this review will provide a practical summary of new developments in this rapidly evolving field and practical advice to providers considering LAAO for their patients. A focus will be placed on understanding the current and emerging indications for LAAO, contemporary data supporting LAAO use, and antithrombotic strategies. Emerging indications for LAAO including concomitant LAAO during structural heart procedures such as transcatheter aortic valve replacement (TAVR), mitral transcatheter edge-to-edge repair (M-TEER), transcatheter mitral valve replacement (TMVR), and concomitant LAAO at the time of electrophysiologic procedures including NVAF ablation will be highlighted. We will further discuss practical considerations regarding LAAO planning and procedural technique, including the use of computed tomography (CT) for screening, and intracardiac echocardiography (ICE) as an alternative to transesophageal echocardiography (TEE) for procedural guidance.

## Current Indications for LAAO

**Figure 1:**
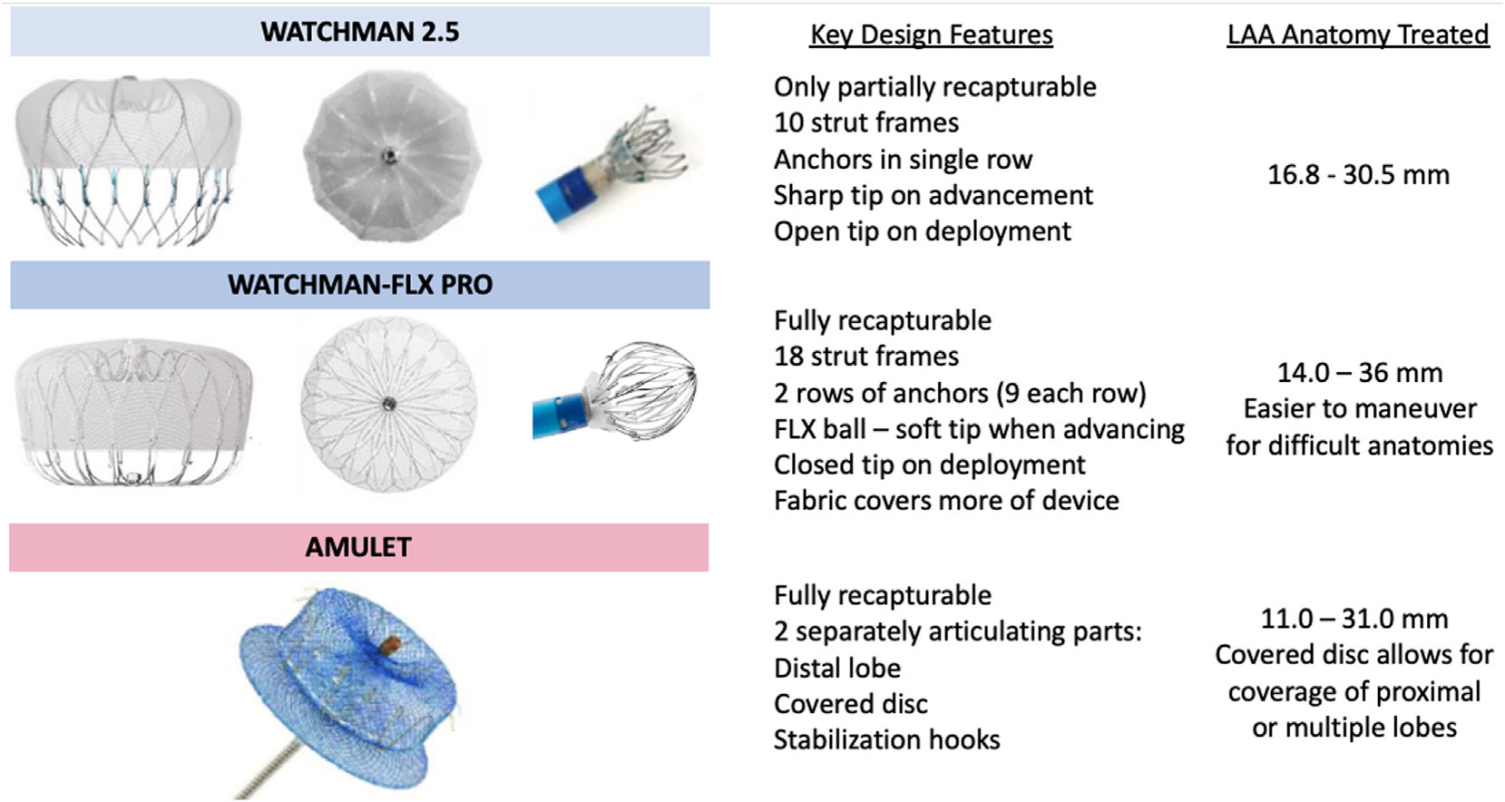
Key design features of the WATCHMAN 2.5, WATCHMAN-FLX, and Amulet devices Abbreviation: LAA, left atrial appendage.

International professional societal guidelines and expert consensus documents give percutaneous LAAO a Class IIA recommendation for prevention of ischemic stroke and systemic embolism in patients at risk for thromboembolic complications from NVAF and have a contraindication to long-term anticoagulation due to a nonreversible cause.^1^^,^^2^^,^^4^^,^^15^^,^^16^ It is also reasonable (Class IIb) in patients with high risk of major bleeding on OAC based on patient preference, with careful consideration of procedural risk. The currently approved indications for use of LAAO in the United States are provided in Table 1, and approved devices in the United States are shown in Figure 1. Indications for use are similar in the European Union, with the important exception that LAAO is approved in a broader segment of patients including those with true contraindications for OAC,^15^ while in the United States, patients must be able to tolerate short-term OAC or DAPT following the procedure.^1^

**Table 1:** Current indications for percutaneous LAAO in the United States

LAAO is indicated to reduce the risk of thromboembolism from the LAA in patients with NVAF who
1 Are at increased risk for stroke and systemic embolization based on CHADS_2_ or CHADS_2_-VASc score ≥2.
2 Are deemed suitable for at least short-term antithrombotic therapy post-LAAO.
3 Have an appropriate rationale to seek a nonpharmacologic alternative to OAC.
5 No other indication for OAC than AF (e.g., prior VTE, mechanical valve, presence or predisposition to left atrial or left ventricular thrombus).
6 Anatomy appropriate for LAAO.

Abbreviations: AF, atrial fibrillation; LAA, left atrial appendage; LAAO, left atrial appendage occlusion; NVAF, nonvalvular atrial fibrillation or flutter; OAC, oral anticoagulant; VTE, venous thromboembolism.

**Figure 2:**
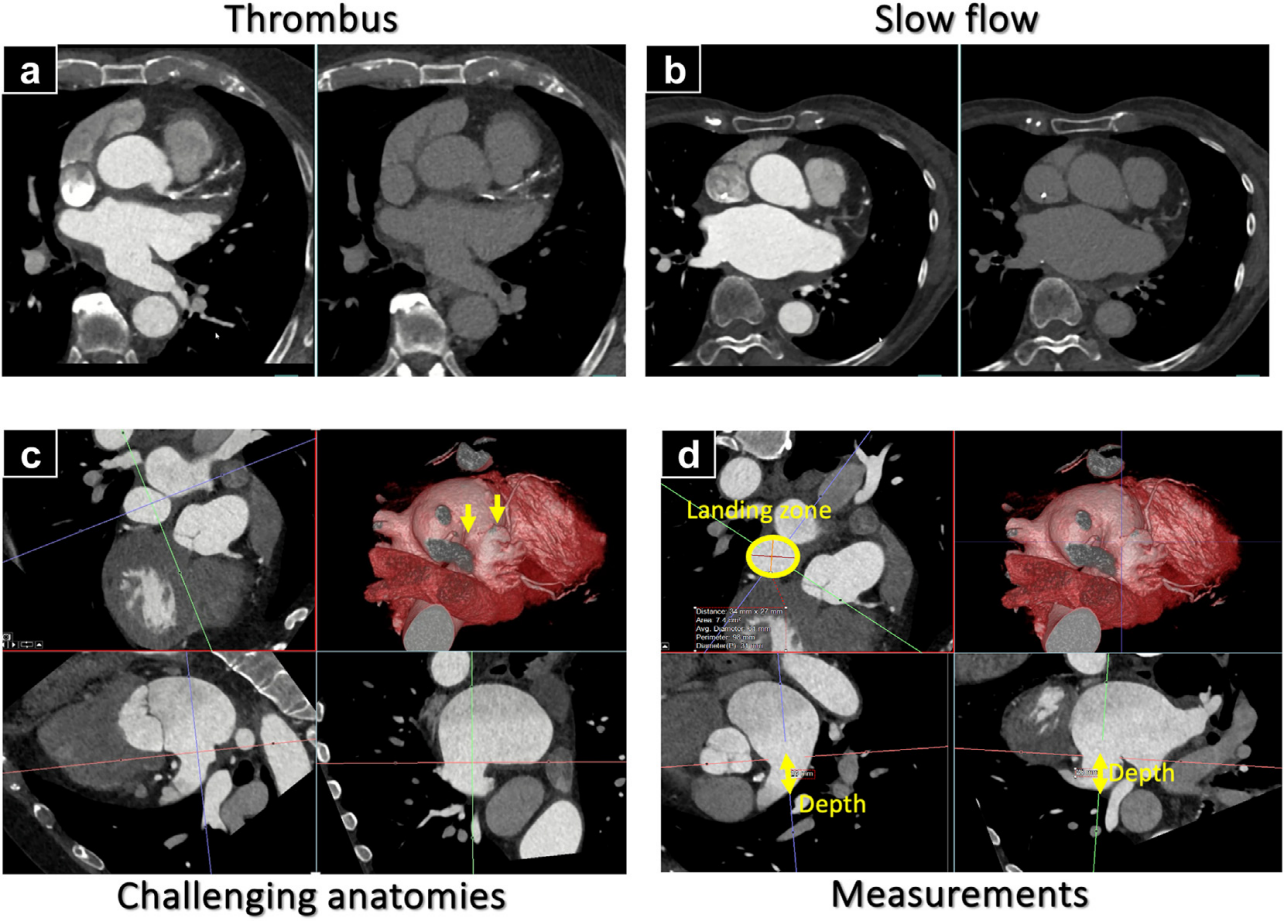
Common anatomic considerations for LAAO. (a) CT imaging of LAA thrombus; (b) slow flow in the LAA. Filling defect that persists with delayed acquisition is likely thrombus (panel A), but if it becomes homogenous, it is likely slow flow (panel B); (c) challenging anatomy with large LAA and multiple lobes; (d) note that given adequate implantation depth, LAAO was still successful in this patient. Videos of panels A-C are included in the supplement Abbreviations: CT, computed tomograph; LAA, left atrial appendage; LAAO, left atrial appendage occlusiony.

Contraindications to percutaneous LAAO are summarized in Table 2. These include the inability to tolerate short-term OAC or antiplatelet therapy post-LAAO, presence of another indication for anticoagulation given the lack of data demonstrating a benefit in addition to OAC, and anatomic exclusion for LAAO.^17^ The most common anatomic exclusions are the presence of thrombus in the LAA or shallow LAA anatomy with multiple lobes, which may not be fully covered by currently available LAAO devices (Figure 2 with videos).

**Table 2:** Contraindications to LAAO

1 Inability to take short-term OAC or antiplatelet therapy post-LAAO.
2 Presence of another indication for OAC than AF. Examples: - Prior VTE - Mechanical valve - Hypercoagulable state - Presence or predisposition to left atrial or left ventricular thrombus
3 Anatomy inappropriate for LAAO based on CT or TEE imaging.
4 Prior surgical excision or clipping of LAA.
5 Valvular AF (i.e., rheumatic heart disease)∗

Abbreviations: AF, atrial fibrillation; CT, computed tomography; LAA, left atrial appendage; LAAO, left atrial appendage occlusion; OAC, oral anticoagulant; TEE, transesophageal echocardiography; VTE, venous thromboembolism.

∗ Given limited data in this patient population.

### Key Randomized Clinical Trials Supporting LAAO

A wealth of data supporting the use of percutaneous LAAO has been amassed from numerous randomized clinical trials (RCTs), observational studies, and required postapproval studies over the past 20 years.

The first LAAO device approved was the WATCHMAN 2.5 (Boston Scientific, Marlboro, Massachusetts), which has since iteratively improved as the WATCHMAN-FLX and WATCHMAN-FLX PRO. The pivotal clinical trials that led to the approval of index device as an alternative to warfarin were PROTECT-AF^18^ and PREVAIL.^8^ A third trial, PRAGUE-17, was published in 2020 and affirmed noninferiority of the WATCHMAN 2.5 and WATCHMAN-FLX or Amulet devices to OAC with a DOAC agent.^13^ A high-yield summary of the key findings of these trials is provided in Table 3.

**Table 3:** Summary of findings from the pivotal RCTs of LAAO

	PROTECT AF	PREVAIL	Amulet IDE	PRAGUE-17
Comparison	WATCHMAN vs. warfarin	WATCHMAN vs. warfarin	Amulet vs. WATCHMAN	WATCHMAN 2.5 or WATCHMAN-FLX or Amulet vs. NOAC (apixaban, rivaroxaban, dabigatran)
Design	Noninferiority	Noninferiority	Noninferiority	Noninferiority
Sample size	2:1 device:control randomization	2:1 device:control randomization	1:1 randomization to Amulet or WATCHMAN 2.5; 1878 patients	201 LAAO vs. 201 NOAC
	463 LAAO vs. 244 warfarin	269 LAAO vs. 138 warfarin		
Patient population	CHADS2 ≥1	CHADS_2_ ≥2 and 1 other risk factor (high-risk)	CHADS_2_ ≥2 or CHA_2_DS_2_-VASc ≥3	CHA_2_DS_2_-VASc ≥3
				HAS-BLED ≥3
Primary Endpoint	Efficacy: composite CVA, SE, CV death	1st co-primary endpoint: composite CVA, SE, CV/unexplained death	Primary safety endpoint: procedure-related complications, all-cause death, major bleeding through 12 mo.	Composite CVA, TIA, SE, CV death, major or nonmajor bleed, procedure/device complications
	Safety: major bleeding, pericardial effusion, device embolization	2nd co-primary endpoint: late ischemic events (CVA or SE ​> ​7 d)	Primary efficacy endpoint: ischemic stroke, SE through 18 mo.	
		3rd co-primary endpoint: early safety events (all-cause death, ischemic CVA, SE, device/procedure complications		
Follow-up	1065 patient-years	18 mo	18 mo	19.9 mo
Key results	Efficacy—WATCHMAN noninferior to warfarin: 3 per 100 patient years vs. 4.9 per 100 patient years (RR 0.35-1.25)	1st co-primary endpoint similar between WATCHMAN (0.064) and warfarin (0.063) but noninferiority not technically met (RR 1.07)	Amulet noninferior to Watchman for safety and efficacy.	No differences in composite (10.99% vs. 13.42%; sHR 0.53-1.31) or any component of composite between LAAO and NOAC
	Safety: 7.4 per 100 patient years vs. 4.4 per 100 patient years (RR 1.01-3.19)	2nd co-primary endpoint of late ischemic events noninferior	Complete LAAO higher with Amulet vs. Watchman (98.9 vs. 96.8%; *p* = 0.003).	
			Procedure complications higher with Amulet (4.5% vs. 2.5%), driven by pericardial effusion.	
Other important points	First generation device; safety/efficacy improved with device improvements	Fewer safety events than PROTECT AF (4.2% vs. 8.2%; *p*-0.004) (3rd co-primary endpoint)	Older generation Watchman 2.5 device used	4.5% LAAO complication rate

Abbreviations: AF, atrial fibrillation; CV, cardiovascular; CVA, cardiovascular accident; IDE, investigational device exemption; LAAO, left atrial appendage occlusion; NOAC, novel oral anticoagulant; RCTs, randomized clinical trials; RR, risk ratio; SE, systemic embolism; sHR, sub-distribution hazard ratio; TIA, transient ischemic attack.

The headline results of these RCTs evaluated Watchman at 1.5 years approximately. Long-term efficacy and safety outcomes after LAAO have remained favorable, and they show that the benefits of LAAO further accumulate over time as a significant reduction of major bleeding events.^9^^,^^14^ In this regard, in a pooled analysis out to 5 years, including 1114 patients treated with WATCHMAN 2.5 in PROTECT-AF and PREVAIL, LAAO had similar rates of composite stroke, systemic embolism (SE), or cardiovascular/unexplained death as warfarin (hazard ratio [HR] 0.820, *p* = 0.27). However, LAAO had less hemorrhagic stroke (HR 0.20, *p* = 0.0022), disabling/fatal stroke (HR 0.45, *p* = 0.03), and postprocedure bleeding (HR 0.48, *p* = 0.0003) than OAC. As a result, cardiovascular death (HR 0.59, *p* = 0.027), and all-cause death (0.73; *p* = 0.035) were lower with LAAO when patients were followed in the long-term.^14^

Data from these RCTs indicate excellent short-, medium-, and long-term results with LAAO compared to OAC with warfarin or DOAC agents. They confirm that the benefits of LAAO accumulate over the lifetime of the patient, including a significant reduction in major bleeding events and a potential reduction in cardiovascular and all-cause mortality over OAC strategies.

### Device Developments: WATCHMAN-FLX Series

Considering these initial RCT data, the US Food and Drug Administration (FDA) approved the WATCHMAN 2.5 device in April 2015. Subsequently, postmarket registry and large observational studies have confirmed the safety and efficacy of this first-generation WATCHMAN 2.5 device.^16^

Recent studies have further shown improved clinical outcomes with the next generation WATCHMAN-FLX device, owing to significant changes in device design that improve the safety of deployment by less risk of trauma to the LAA, better LAA closure rates, and less device-related thrombus (DRT).^19^ Figure 1 provides key features of the design of the WATCHMAN 2.5 as a comparator to the newer WATCHMAN-FLX. Improvements to the WATCHMAN-FLX design include both larger available diameters as well as a shallower device at all sizes, a larger polytetrafluoroethylene cover to reduce metal exposure, an 18 strut frame to improve tissue contact and seal, two rows of 9 anchors, each with a less traumatic design to improve tissue engagement, and a closed distal tip to improve the safety. A key step in the deployment of the WATCHMAN-FLX is formation of the WATCHMAN FLX ball, as in partial deployment, the device forms a minimally traumatic “ball” without any sharp edges and can be advanced safely and maneuvered within the LAA to achieve an optimal position and seal. This increased maneuverability allows for better coverage of challenging anatomies, including selection of the optimal distal lobe and coverage of proximal lobes. The device is fully recapturable at any step of deployment prior to release. The delivery sheath has also been improved to be more maneuverable and is now integrated with the transseptal puncture system as the WATCHMAN Connect.

The PINNACLE FLX study and subsequent data from the NCDR LAAO registry (SURPASS) have confirmed the superiority of the WATCHMAN-FLX device over the prior WATCHMAN 2.5.^16^^,^^19^ PINNACLE FLX was a prospective, nonrandomized registry of 400 patients treated with the WATCHMAN-FLX device and confirmed a 100% LAA closure rate with WATCHMAN-FLX (defined as ≤5 mm peridevice leak [PDL] at 12 months). There was further reduction in DRT compared to rates seen with WATCHMAN 2.5.^1^^,^^15^ In real-world data from the SURPAASS registry of 16,445 patients treated with WATCHMAN FLX, procedural success was excellent at 97.6%. Safety was excellent, with only 0.37% of patients experiencing a device or procedure-related event (all-cause death, stroke/SE, device/procedure complication) and more patients with a complete seal (95% with PDL <3 mm). WATCHMAN FLX patients also had comparatively lower mortality (0.91%), ischemic stroke (0.28%), pericardial effusion requiring intervention (0.51%), and DRT (0.23%).^20^

Considering these data, FDA approval of the WATCHMAN FLX device was obtained in July 2020. The FLX device has since been used in nearly 190,000 of the total 300,000 WATCHMAN procedures completed. In September 2023, the WATCHMAN FLX Pro was released, consisting of an enhanced polymer coating designed to promote faster healing and endothelization of the device surface, markers for better visualization during fluoroscopic deployment, and a new 40 mm size device to treat larger LAA diameters (Figure 1).

### Recent Device Developments: Amulet

The Amplatzer Amulet device (Abbott) consists of two elements: a distal lobe that sits in the LAA and a disc that fully covers the LAA ostium (Figure 1). A single row of stabilization hooks protrudes from the distal lobe to anchor the device in the LAA. The anchoring lobe is flexible and conforms to most LAA anatomies within the range specified for size of device. The anchoring lobe and covering disc articulate separately, which allows for treatment of complex LAA anatomies with multiple lobes or proximal lobes that may be difficult to cover.

In 2021, the FDA approved the Amplatzer Amulet device for the same indications as WATCHMAN. This was based on the results of the Amulet IDE trial, which randomized 1878 high-risk patients to LAAO with Amulet vs. WATCHMAN 2.5 and found the Amulet device was noninferior to WATCHMAN 2.5 regarding safety and efficacy of stroke prevention.^11^ There was, however, slightly superior success for LAA occlusion with Amulet vs. WATCHMAN 2.5 (98.9% vs. 96.8%, *p* = 0.003 for superiority), with more patients with a complete seal (63% vs. 46%), though there was no significant difference in significant PDL >5 mm at 45 days (1% vs. 3%). There was slightly more pericardial effusion up to 10 days with the Amulet device, the time course for which differed in comparison to the WATCHMAN 2.5, for which most of these events occurred within 24 ​hours. It should be noted that DAPT was used in the Amulet group, while patients with WATCHMAN 2.5 were treated with OAC ​+ ​​aspirin as per the FDA instructions for use.

Data from the 5499 patients who underwent Amulet LAAO in the NCDR LAAO Registry indicates a slightly lower major adverse event rate than the IDE trial of 5.7% out to 45 days, mainly driven by major bleeding and pericardial effusion.^21^ These findings are supported by results from a large multicenter registry with 2-year follow-up,^10^ as well as long-term follow-up data from the PRAGUE-17 trial to 4 years.^9^

### Data Comparing LAAO Devices

Given the results of the Amulet IDE, which showed superiority to WATCHMAN 2.5 with regard to seal and PDL, there was impetus to compare the Amulet device to the newer WATCHMAN FLX device. The SWISS-APERO trial was a 3-way comparison of LAAO with the Amulet (111 patients), WATCHMAN 2.5 (25 patients), or WATCHMAN FLX (85 patients) in eight European centers.^22^ The trial found no difference in the primary endpoint of composite crossover to a nonrandomized device during the procedure or residual LAA patency detected by CT at 45 days between devices. Likewise, there was no difference in composite cardiovascular death, stroke, or SE between Amulet or WATCHMAN patients. However, similar to the Amulet IDE, there was more major bleeding and pericardial effusion with Amulet than WATCHMAN (9.0% vs. 2.7%; *p* = 0.047). Small PDLs were more frequent with TEE in WATCHMAN patients but were more common with cardiac computed tomography angiography with Amulet. Nevertheless, there were no clinically significant PDLs >5 mm. Data from large postapproval registries of Amulet and WATCHMAN FLX are shown in Table 4.^19^, ^20^, ^21^ The takeaway message is that either WATCHMAN FLX or Amulet achieve success, and either device is an excellent choice for LAAO.

**Table 4:** Summary of registry data on contemporary LAAO devices

	Emerge LAA	PINNACLE FLX	SURPAASS
Comparison	Amulet postapproval registry	WATCHMAN FLX postapproval registry	WATCHMAN FLX outcomes from NCDR LAAO Registry
Sample size	5499 patients	400 patients	16,446
Implant success	95.8%	100%	97.6%
Complete occlusion at 45 d	87.1%	100%	
Major adverse events at 45 d			
PE requiring intervention	1.9%	0.7%	0.5%
Device embolization	0.2%	0%	0.03%
Stroke	0.3%	0.7%	0.4%
Death	1.0%	0.5%	0.9%
Other important points	Less PE req intervention at 45 d with improved operator experience (1.5 vs. 2.3%; *p* = 0.012).	0.5% primary safety event; significantly below performance goal 4.2%.	Similar event rates as PINNACLE FLX. Early results; full data not yet published.

Abbreviations: LAA, left atrial appendage; LAAO, left atrial appendage occlusion; PE, pericardial effusion.

### Ongoing Pivotal RCTs

Two large RCTs will further define the role and safety of LAAO in clinical practice as a first-line therapy for stroke prevention in NVAF. The CHAMPION AF trial (NCT04394546) will randomize 3000 patients with NVAF and CHADS2-VASc ≥2 for men or ≥3 for women to LAAO with WATCHMAN FLX vs. DOAC.^23^ Patients will be evaluated for ischemic and bleeding outcomes and followed for 5 years. Importantly, warfarin is not permitted, and elevated bleeding risk is not required for entry; this trial will thus assess a “WATCHMAN-first” approach as an alternative to OAC. The CATALYST trial (NCT04226547) will similarly randomize 2650 patients to Amulet vs. DOAC and follow patients for ischemic and bleeding events out to 3 years. The LAAOS-4 trial (NCT05963698) will investigate whether endovascular LAAO devices prevent ischemic stroke or systemic embolism in atrial fibrillation (AF) patients who remain at high risk of stroke despite OAC. It will randomize 4000 patients to either a Watchman device along with OAC vs. OAC.

## Antithrombotic Strategies Post-LAAO

The optimal postimplant antithrombotic regimen remains debatable, as thrombotic risks should be balanced against bleeding risks. DRT is an infrequent complication post-LAAO but may predispose to stroke and require prolonged anticoagulation to resolve (Figure 2). Benchtop studies indicate that WATCHMAN and Amulet devices completely endothelialize within 30 to 90 days postimplantation, during which time they may be predisposed to DRT.^24^ In appreciation of this, early studies of WATCHMAN 2.5 utilized warfarin in addition to low-dose aspirin for 45 days postprocedure. In combined analysis from PREVENT-AF and PREVAIL, DRT was low at 3.7% overall and only 0.9% during the first 45 days postprocedure while warfarin was being used.^25^ Analysis from the NCDR LAAO registry suggests that combination of warfarin and antiplatelet agents appears to increase bleeding risk without having a beneficial impact on thromboembolic risk.

As warfarin was the comparator utilized in PROTECT-AF and PREVAIL trials,^8^^,^^18^ and DOAC agents (apixaban, rivaroxaban, and dabigatran) were not yet available in the US market, warfarin was the only OAC advised by the FDA for post-LAAO anticoagulation after initial US approval.

### DOACs vs. Warfarin Post-LAAO

After approval of DOAC agents, questions were raised regarding the efficacy of LAAO compared to DOACs for post-LAAO prevention of DRT. The PRAGUE-17 trial answered this question by randomizing 402 patients at high-risk for stroke or SE as well as high-risk for bleeding to LAAO with WATCHMAN 2.5 or WATCHMAN-FLX or the Amulet device vs. DOAC (most frequently apixaban).^9^^,^^13^ The study found equivalent ischemic and bleeding events with both strategies out to 20 months of average follow-up (Table 3), with low rates of DRT in support of using DOACs post-LAAO in appropriate patients. The safety of DOACs for post-LAAO anticoagulation has further been demonstrated in real-world data from large US and multinational registries including SURPASS and SWISS-APERO, which in conjunction suggest DRT rates of 0% to 1.3% and lower bleeding risk compared to warfarin, similar to general NVAF trials of DOAC vs. warfarin.^20^^,^^22^ Given their ease of use and safety profile, DOACs have now been widely embraced post-LAAO and are the comparator group for the large ongoing RCTs CHAMPION AF and CATALYST (as mentioned above).

### DAPT and Alternative Regimens of OAC Post-LAAO

Data comparing the use of post-LAAO anticoagulation vs. DAPT generally suggest similar safety with regard to bleeding events, but potentially higher rates of DRT within 45 days in patients on DAPT.^26^ This may especially be the case in patients who have high on-treatment platelet reactivity with clopidogrel.^27^ Accordingly, while a DAPT strategy is approved in the US, it should in general be reserved for patients with high bleeding risk or true contraindications to OAC, as more data are needed on short- and long-term DRT.

In the subset of patients at extreme risk for bleeding, single antiplatelet therapy or no-AC post-LAAO has been explored as an alternative. Small studies have shown mixed results, with DRT rates of 0% to 7% with a SAPT-only strategy.^28^, ^29^, ^30^ Similarly, while certain studies suggest a strategy of no-AC and no-antiplatelet agents may be safe, data are scant, and some studies indicate a DRT rate >10% in such patients.^31^^,^^32^ There are several ongoing clinical trials evaluating the safety of DAPT, SAPT, and reduced-dose AC post-LAAO.

### Summary of Post-LAAO Antithrombotic Recommendations

**Figure 3:**
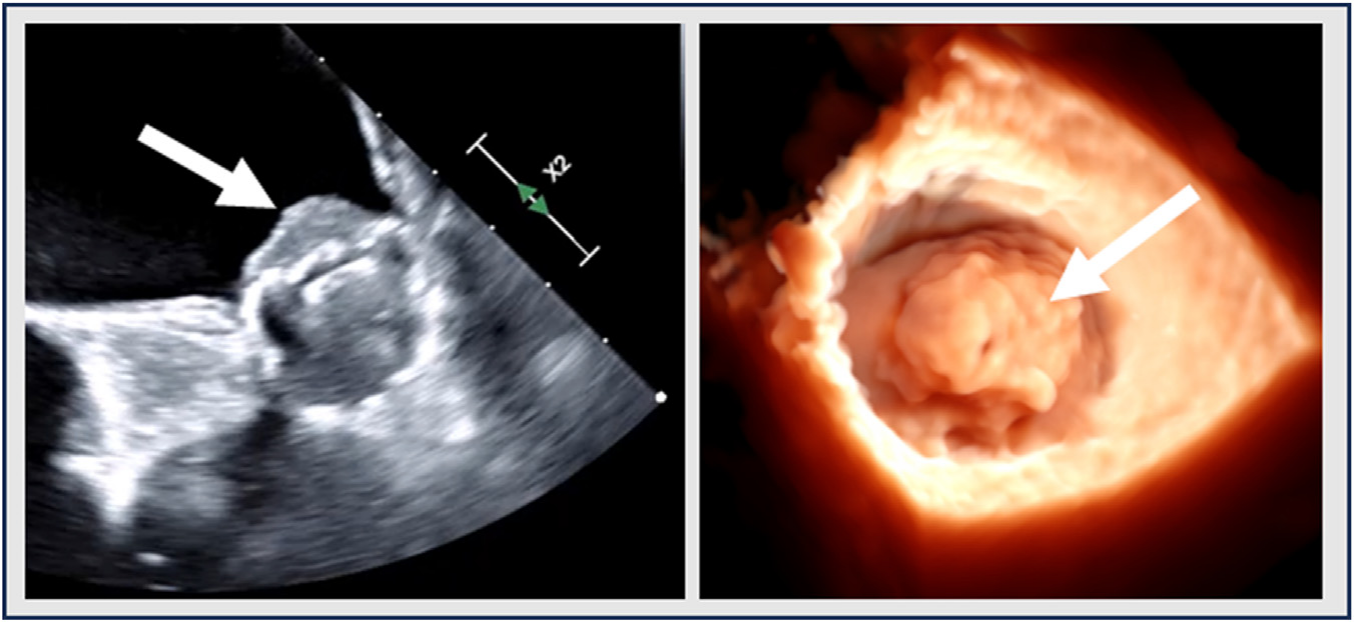
Device-related thrombus (DRT). The 2D and 3D TEE images of DRT attached to the LA surface of a Watchman 2.5 device Abbreviations: 2D, two-dimensional; 3D, three-dimensional; LA, left atrial; TEE, transesophageal echocardiography.

**Figure 4:**
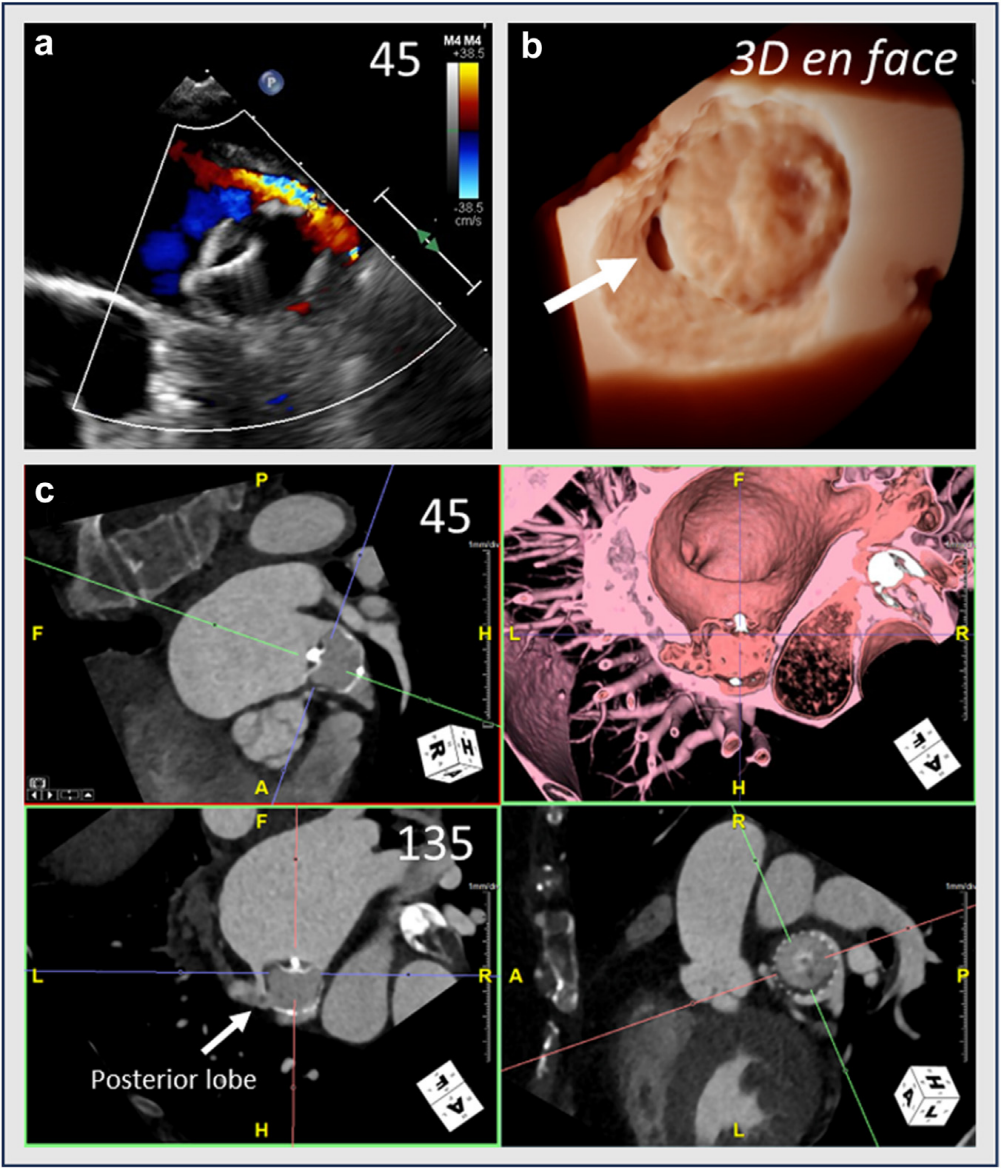
TEE and CT images of a patient with peridevice leak (PDL). (a) TEE of a significant PDL >5 mm visualized posteriorly on color flow Doppler with Nyquist decreased to visualize lower velocity flow. (b) 3D TEE *en face* view of the Watchman device with a peridevice gap visualized posteriorly (arrow). (c) Cardiac CT demonstrating the leak communicates with a posterior lobe Abbreviations: 3D, three-dimensional; CT, computed tomography; TEE, transesophageal echocardiography.

In the United States, LAAO is only approved in patients who can tolerate antithrombotic therapy for 6 months post-LAAO as per 1 of 2 antithrombotic strategies as summarized in Table 5.^1^^,^^2^ If utilizing an anticoagulation strategy, warfarin or DOAC ​+ ​low-dose aspirin of 81 to 100 mg daily is advised for 45 days post-LAAO. At 45 days, a TEE or computed tomography angiography is obtained to assess for significant PDL (defined as a leak >5 mm) or DRT (Figures 3 and 4). If no significant PDL or DRT is present, OAC can be stopped and clopidogrel 75 mg daily taken for the duration of 6 months (days 46-180). At 6 months, clopidogrel is stopped and low-dose aspirin is continued indefinitely. If significant PDL or DRT is found at 45 days, OAC is not stopped given continued risk of SE.

**Table 5:** Approved antithrombotic strategies post-LAAO

Anticoagulation strategy	
Days 1-45:	Aspirin 81-100 mg daily ​+ ​OAC (warfarin or NOAC)
Days 46-6 mo:∗	Aspirin 81-100 mg daily ​+ ​clopidogrel 75 mg daily
6 mo-onward:	Aspirin 81-100 mg daily
Dual-antiplatelet strategy	
Days 1-45:	Aspirin 81-100 mg daily ​+ ​clopidogrel 75 mg daily
Days 46-6 mo:∗	Aspirin 81-100 mg daily ​+ ​clopidogrel 75 mg daily
6 mo-onward:	Aspirin 81-100 mg daily

Abbreviations: CT, computed tomography; DAPT, dual antiplatelet therapy; LAA, left atrial appendage; LAAO, left atrial appendage occlusion; NOAC, novel oral anticoagulat; OAC, oral anticoagulant; PDL, peridevice leak; TEE, transesophageal echocardiography.

∗ Assuming adequate LAA closure at a 45-d assessment by TEE or cardiac CT. If there is inadequate LAA closure (PDL >5 mm), OAC should not be stopped, or OAC should be resumed if a DAPT strategy is utilized.

If utilizing a DAPT strategy, low-dose aspirin of 81 to 100 mg ​+ ​clopidogrel 75 mg daily is utilized postprocedure. At 45 days, a TEE or CTA is similarly obtained, and if significant PDL or DRT is not present, DAPT is continued for the duration of 6 months (the full 180 days). Then, clopidogrel is stopped, and low-dose aspirin is utilized indefinitely. If there is significant PDL or DRT at 45 days, OAC should be started and clopidogrel discontinued. Note that TEE may be particularly useful for evaluation of unusual leaks, such as defects through the device fabric (Figure 3).

In the European Union, LAAO is approved for patients with true contraindications for OAC or DAPT, but are able to tolerate single antiplatelet therapy with aspirin or a P2Y12i.^15^ There are numerous ongoing prospective studies and RCTs in this space, which will further inform optimal antithrombotic strategies and treatment durations after LAAO.

### Management of Device-Related Thrombus

The pooled incidence of DRT is approximately 3.8% (3.4% in Watchman 2.5 device, 4.8% in the Amulet device, and 1.8% in the Watchman FLX device). Although there is no evidence of superiority of one device vs. another with respect to DRT, the timing of DRT formation is different between these devices. DRTs with Watchman devices often occur after 45 days, while DRTs with the Amulet device often occur within 45 days. This may be related to the device or the postimplant antithrombotic regimen used with these devices.

Clinical predictors of DRT include older age, history of prior ischemic event, permanent AF, high CHADS2-VASC score, or presence of renal failure. Echocardiographic predictors include large left atrial (LA), large LAA appendage, presence of spontaneous echo contrast, and reduced left ventricular systolic function. Procedure/device-related predictors include procedure-related pericardial effusion and deep-seated device.

Although the association between DRT and thromboembolic events has not been consistent, the use of anticoagulation is often recommended once DRT is diagnosed and preferably continued until the complete resolution of DRT. Serial screening may be warranted to check for recurrence, which may warrant reinitiation of long-term anticoagulation.

Watchman FLX PRO device was recently introduced and has a fabric membrane coated with a hemocompatible fluoropolymer coating. In a preclinical canine study, this coating resulted in lower incidence of thrombus formation and local inflammation and higher incidence of complete endothelialization. The HEAL-LAA trial is currently underway to collect real-world data on WATCHMAN FLX Pro.

### Management of Significant PDL Post-LAAO

The mechanism of PDL may be due to a leak at the edge of the device, an uncovered lobe or proximal LAA tissue, or through the fabric itself. While the device reendothelializes, a small fabric leak is commonly seen; this does not appear to have clinical significance and often resolves within 90 days.

Data on the prognostic impact of PDLs on thromboembolic risk is mixed. Initial data from the PROTECT-AF trial did not find any relationship between the severity of PDL and the risk of ischemic stroke or systemic embolization. However, the NCDR LAAO registry showed an increased risk, especially in patients with PDL at 1 year postimplant. In view of these findings, it may be reasonable to close PDL, especially in severe (≥5 mm) or symptomatic (ischemic event) (PDL ≥3 mm), especially when it is connected to an uncovered proximal lobe.

The incidence of PDL in major clinical trials and registries is heterogeneous due to a lack of consensus on method of leak detection (e.g., TEE vs. CT) and threshold for what is considered potentially significant. Real-world data of 51,333 patients treated with the earlier generation WATCHMAN 2.5 in the NCDR LAAO registry indicate that while the rate of large PDL >5 mm is low (0.7%), many patients have small leaks 1 to 5 mm (25.8%).^33^ Data from the PINNACLE FLX registry indicate a lower rate of any PDL at 45 days (17.4%) and 1 year (10.5%).^12^^,^^19^ While the pivotal clinical trials leading to LAAO approval utilized >5 mm as a cut-off for clinically significant PDL, recent data suggest that even small PDLs of 1 to 5 mm may slightly increase the risk of stroke and SE, and thus pursuit of a complete closure should be the goal in every patient.^33^

**Figure 5:**
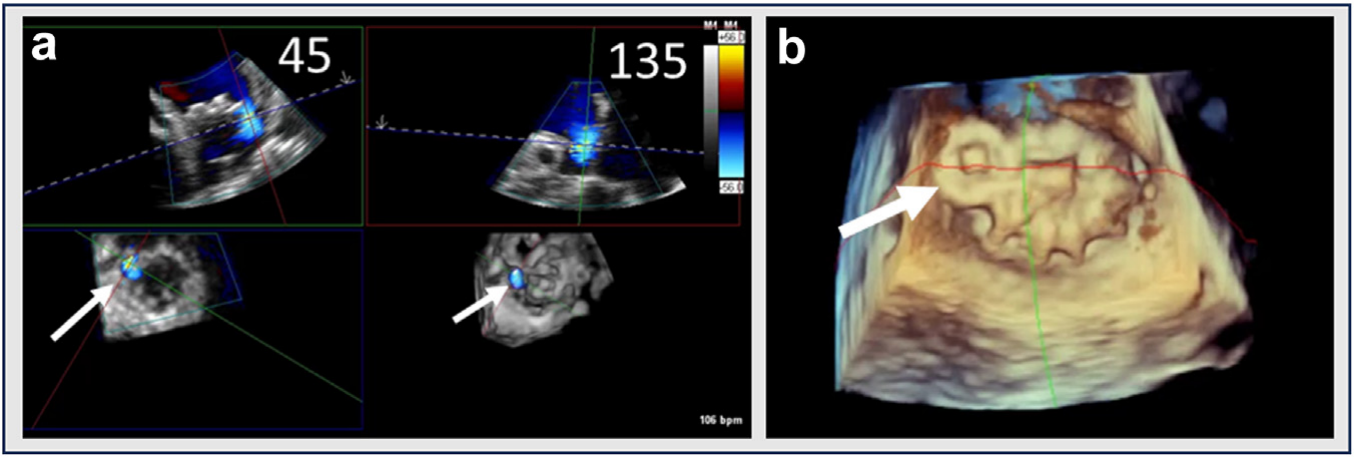
PDL closure with an AVP 2 device. (a) Significant PDL was identified by TEE (similar to the patient in Figure 3). (b) The PDL was wired and closed with a single 12 mm AVP 2 device (arrow), which is deliverable through a standard 5F catheter Abbreviations: AVP, amplatzer vascular plug; PDL, peridevice leak; TEE, transesophageal echocardiography.

In patients with a PDL >5 mm, catheter-based closure of focal PDLs is feasible in many cases and may more comfortably liberate patients from OAC. Options to address PDL include catheter-based introduction of plugs (Figure 5), occluders, coils, radiofrequency ablation, or extraction of the index device, and reimplantation with different positioning or consideration of a different size or device type.^34^ A thorough evaluation with CT and/or TEE and an understanding of the mechanism of PDL are essential for assessing the appropriate approach. If plug-based PDL closure is considered, it is encouraged that the operator wait at least 90 days post-LAAO to allow for adequate time for the device to endothelialize, which may reduce the risk of device embolization.

## Emerging Indications for LAAO

As the field of LAAO has matured, procedural risks have fallen, and outcomes have continued to improve. Long-term follow-up from RCTs indicates a lower risk of major bleeding events after LAAO than OAC, which may reduce mortality and lower health care expenditures. Liberation from OAC has several advantages, and if given the option, many patients may choose LAAO instead. Taken in conjunction, a compelling case can be made for LAAO as an alternative to OAC in all appropriate patients, regardless of bleeding risk. Ongoing studies of NVAF patients at low risk of SE (i.e., CHADS2-VASc ≥1) and patients at all levels of bleeding risk stand poised to position LAAO as an alternative to OAC in all-comers.

## Concomitant LAAO During Structural and Electrophysiology Procedures

The combination of LAAO with other structural heart and electrophysiology (EP) procedures is attractive for several reasons. While LAAO is typically performed as an isolated procedure, many patients referred for ablation of NVAF or valvular heart interventions for aortic or mitral valve (MV) disease also have an indication for LAAO as well. Combination LAAO may have specific advantages, especially during procedures that share common procedural steps such as the need for femoral venous access, transseptal puncture, transesophageal echocardiography (TEE) or ICE, monitored, or general anesthesia care. This may further allow for gains in efficiency, shorter length of stay compared to two separate procedures, reduced health care costs, and improved patient satisfaction. Combining LAAO at the time of such procedures may be viewed as a strategy similar to LAA clipping or ligation offered at the time of open heart surgery for another reason.

Illustrating national trends and the safety of concomitant LAAO, in a recent study of 88,910 patients, the volume of concomitant LAAO rose from 2016 to 2020, though it is still uncommon and represents only ∼1.4% of LAAO procedures.^35^ Most concomitant LAAO procedures were done at the time of NVAF ablation (73.2%) or TAVR (15.5%). In a propensity-score matched comparison, while there was some heterogeneity regarding outcomes owing to the nature of the “primary” procedure, there were overall similar risks between isolated cardiac procedures and concomitant procedures (to include LAAO), with no difference in in-hospital mortality, stroke, acute kidney injury, major bleeding, vascular injury, or length of stay. While there were higher costs associated with concomitant LAAO, there were lower costs compared to LAAO with sequential procedures, speaking to the economic benefits of this approach.^35^

There are several recently completed and ongoing studies evaluating concomitant LAAO in combination with another procedure.

### TAVR ​+ ​LAAO

**Figure 6:**
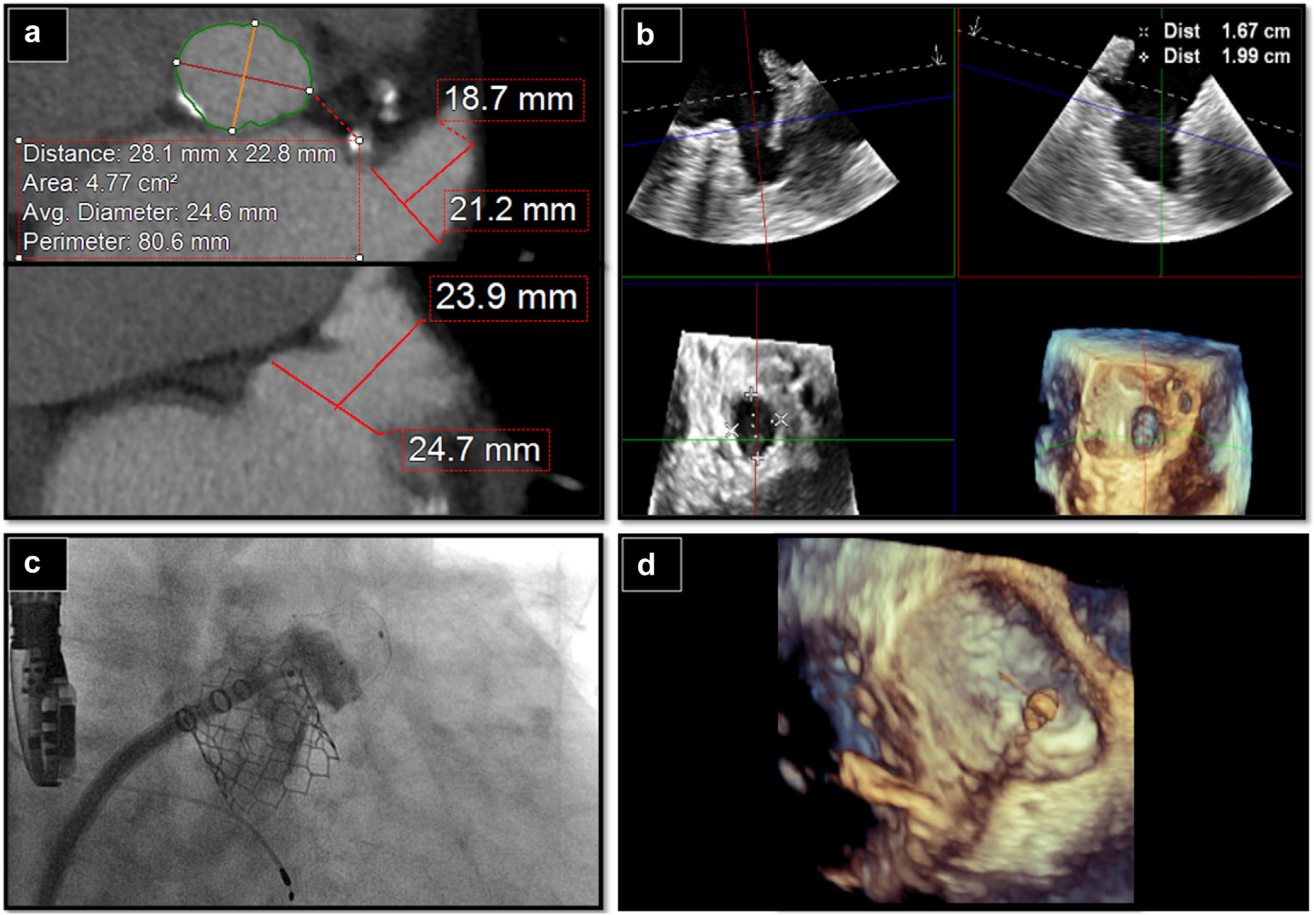
Example of combined TAVR ​+ ​Watchman LAAO. (a) Use of the pre-TAVR CT scan to measure both the AV annulus (left top panel) and LAA for Watchman placement (right top and bottom panel); (b) correlation of CT and TEE sizing; (c) fluoroscopic images of Watchman placement in RAO caudal view, separating TAVR and Watchman devices for visualization; (d) final TEE following successful 27 mm Watchman FLX Pro Abbreviations: AV, aortic valve; CT, computed tomography; LAA, left atrial appendage; LAAO, left atrial appendage occlusion; RAO, right anterior oblique, TAVR, transcatheter aortic valve replacement; TEE, transesophageal echocardiography.

Approximately 15% to 40% of patients with severe, symptomatic aortic stenosis also have NVAF,^36^ of which >90% have a CHADS2-VASc score >3,^37^ and >5% of patients have major bleeding within 30 days to 1 year.^37^ The combination of TAVR ​+ ​LAAO is appealing, as this mimics the common surgical approach of surgical aortic valve replacement ​+ ​LAA clipping or ligation and may address the need for long-term prevention of stroke and minimize the risks of bleeding by eliminating the need for OAC. LAAO at the time of TAVR may further reduce procedural risks in that it reduces the need for repeat venous access and utilizes the same femoral access site utilized for TAVR, and the pre-TAVR CT scan may be utilized for LAAO planning as well (Figure 6).

The WATCH-TAVR trial was the largest RCT to evaluate the safety and effectiveness of a concomitant LAAO procedure.^38^ WATCH-TAVR randomized 349 patients with severe aortic stenosis 1:1 to TAVR ​+ ​LAAO with Watchman 2.5 vs. TAVR ​+ ​medical therapy with Warfarin or DOAC. The incremental LAAO procedure took 38 ​minutes and utilized 119 mL of contrast compared to 70 mL for TAVR alone. At 24-month follow-up, 13.9% compared to 66.7% of patients on any OAC, were comparing TAVR ​+ ​LAAO vs. TAVR ​+ ​medical therapy. TAVR ​+ ​LAAO was noninferior to TAVR ​+ ​medical therapy for the composite primary endpoint of all-cause mortality, stroke, and major bleeding at any time point (22.7 vs. 27.3 events per 100 patient-years; *p* ​<0.001 for noninferiority), illustrating the safety of the combined approach. Further, and consistent with other longitudinal data series, only two-thirds of patients in the medical therapy arm were in fact prescribed/adherent to OAC therapy at 24 months.

### Concomitant LAAO Following TEER or TMVR

**Figure 7:**
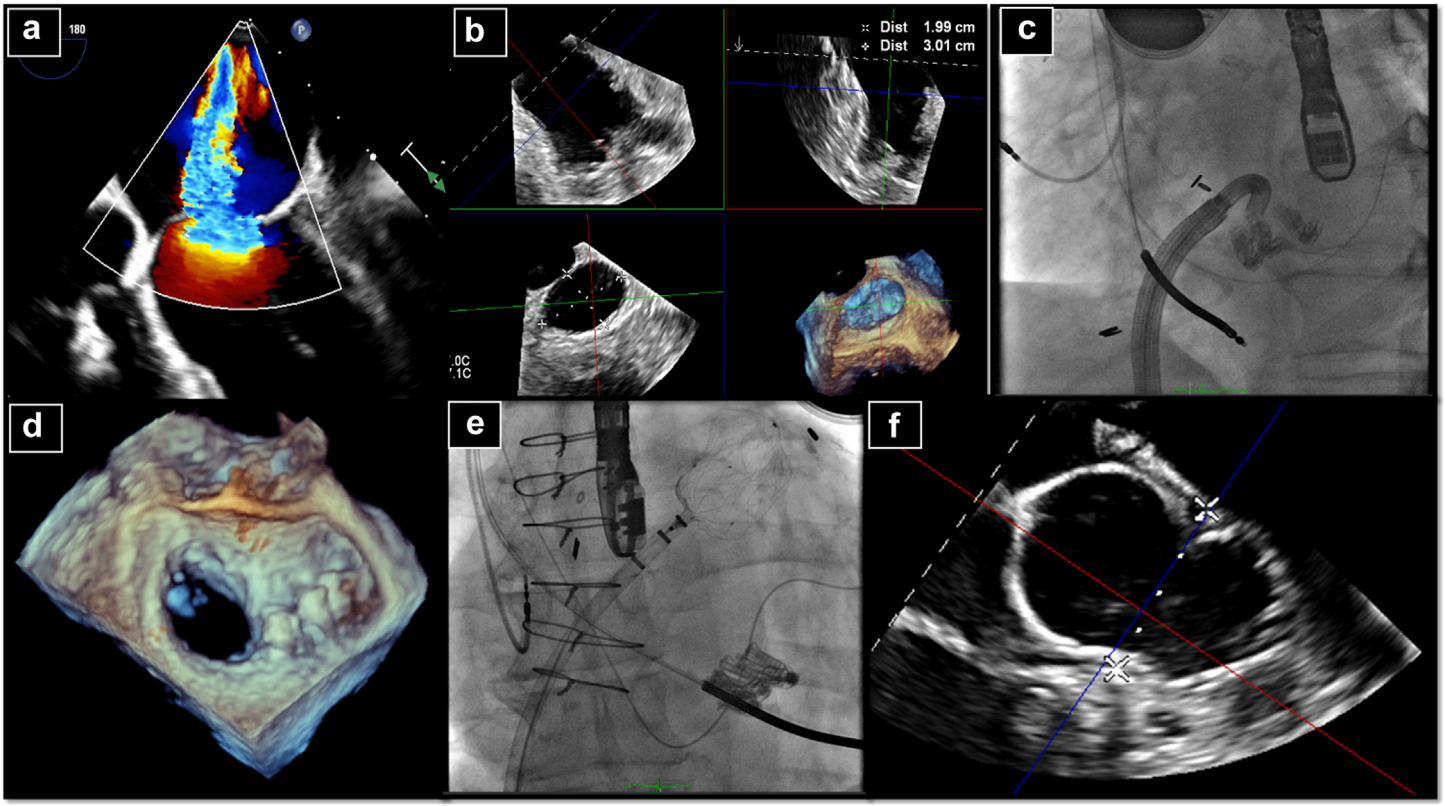
Example of combined M-TEER ​+ ​LAAO. (a and b) Use of TEE prior to M-TEER to assess severity and mechanism of MR and size for Watchman placement; (c and d) placement of three Edwards Pascal/ACE devices; (e and f) successful placement of Watchman FLX Pro device via the same TSP and femoral venous access site used for M-TEER, using fluoroscopy and TEE for guidance Abbreviations: LAAO, left atrial appendage occlusion; M-TEER, mitral transcatheter edge-to-edge repair; MR, mitral regurgitation; TEE, transesophageal echocardiography; TSP, transseptal puncture.

Structural interventional procedures that require LA access have increased in volume significantly in recent years as M-TEER and TMVR options have continued to proliferate.^35^ Concomitant LAAO at the time of mitral structural procedures has several clear advantages including use of the transseptal puncture, general anesthesia, and TEE or ICE already used for the case.^35^^,^^38^ The large-bore venous sheaths and LA access required for these cases lend themselves well to efficient LAAO and eliminates many of the risks of the procedure including TSP, as mentioned (Figure 7). While the septal puncture location differs (superior/posterior for TEER, inferior/posterior for LAAO), the M-TEER TSP can often still be utilized for LAAO. Additionally, steerable guide catheters (WATCHMAN TruSteer and Amplatzer Steerable Delivery Sheath) allow a more liberal TSP location for LAAO.

There are limited data on the safety and efficacy of percutaneous LAAO in patients with significant MV disease, as these patients have been excluded from pivotal clinical trials to date, though it is likely safe and effective.^35^^,^^38^^,^^39^ In a recent analysis of a large administrative database including 51,540 patients, there were similar rates of post-LAAO bleeding, stroke, and mortality in patients with MV disease compared to those without.^40^ Further, treatment of the MV disease may further reduce the risks of stroke and other complications from NVAF. There are ongoing studies of combination LAAO at time of M-TEER, which should better inform this strategy.

### Practical Considerations for Combined Structural/EP ​+ ​LAAO Procedures

As structural and EP procedures expand in volume and scope, careful consideration should be given to the strategy used in patients who may be at risk for complications from creation of an iatrogenic atrial septal defect (iASD),^41^ as well as the need to reaccess the LA for future procedures.^42^ The iASD created from most procedures that require LA access is typically 4.5 to 10 mm in size, depending on the procedure. This may create a temporary intraatrial shunt, which usually heals within several months postprocedure without intervention.^43^ Severe right ventricular dysfunction, severe tricuspid regurgitation, and/or severe pulmonary hypertension have traditionally been considered relative contraindications for LAAO placement as creation of the iASD may cause right to LA shunting. Rather than avoid LAAO in these patients, LAAO may still be considered, though the operator should be prepared to close the iASD if needed.^42^ Typical devices used for this purpose include the GORE Cardioform Septal Occluder (25 mm), GORE ASD Occluder (27 mm), or Amplatzer device. If there is likelihood that the interatrial septum may need to be closed after an LA procedure (i.e., M-TEER or NVAF ablation), consideration should be given to LAAO at the conclusion of the procedure, as accessing the septum around or through a closure device may be possible but challenging.

### AF Ablation ​+ ​LAAO

Concomitant AF ablation and LAAO have been extensively described as a safe and feasible alternative to sequential procedures based on registry and small prospective studies.^44^ Yet, practice patterns in the United States have not adopted this approach primarily due to reimbursement constraints. As the first large-scale randomized trial of this concomitant treatment strategy, the pending results of the OPTION trial (NCT03795298) will reveal whether LAAO with WATCHMAN FLX is an effective and safe alternative to OAC following percutaneous catheter ablation for NVAF.^45^ A total of 1600 patients were randomized 1:1 to catheter ablation ​+ ​WATCHMAN FLX at time of the procedure (concomitant) or between 90-180 days prior to randomization (sequential). Control patients continued DOAC for the duration of the study.

In addition to clarifying the role of LAAO in lieu of OAC in patients with low risk of bleeding and moderate to high risk of stroke, our hope is that the trial (if supportive of the combined treatment) will also introduce a shift in cost/reimbursement strategies that will allow operators to provide concomitant therapy to their patients.^35^

In the interim, traditional candidates for concomitant procedures are those who would benefit from ablation for sinus rhythm maintenance and withdrawal of OAC due to bleeding risk; however, patients with electrical isolation or substantial electrical delay of the LAA should strongly be considered for LAAO.^46^ Electrical isolation occurs in the setting of extensive LA flutter ablation or during deliberate LAA isolation when identified as a trigger for AF. LAAO should be considered given that the risk of embolic stroke in this patient population is elevated beyond their calculated stroke risk even when in sinus rhythm.

### Practical Considerations for Concomitant AF Ablation and LAAO

**Figure 8:**
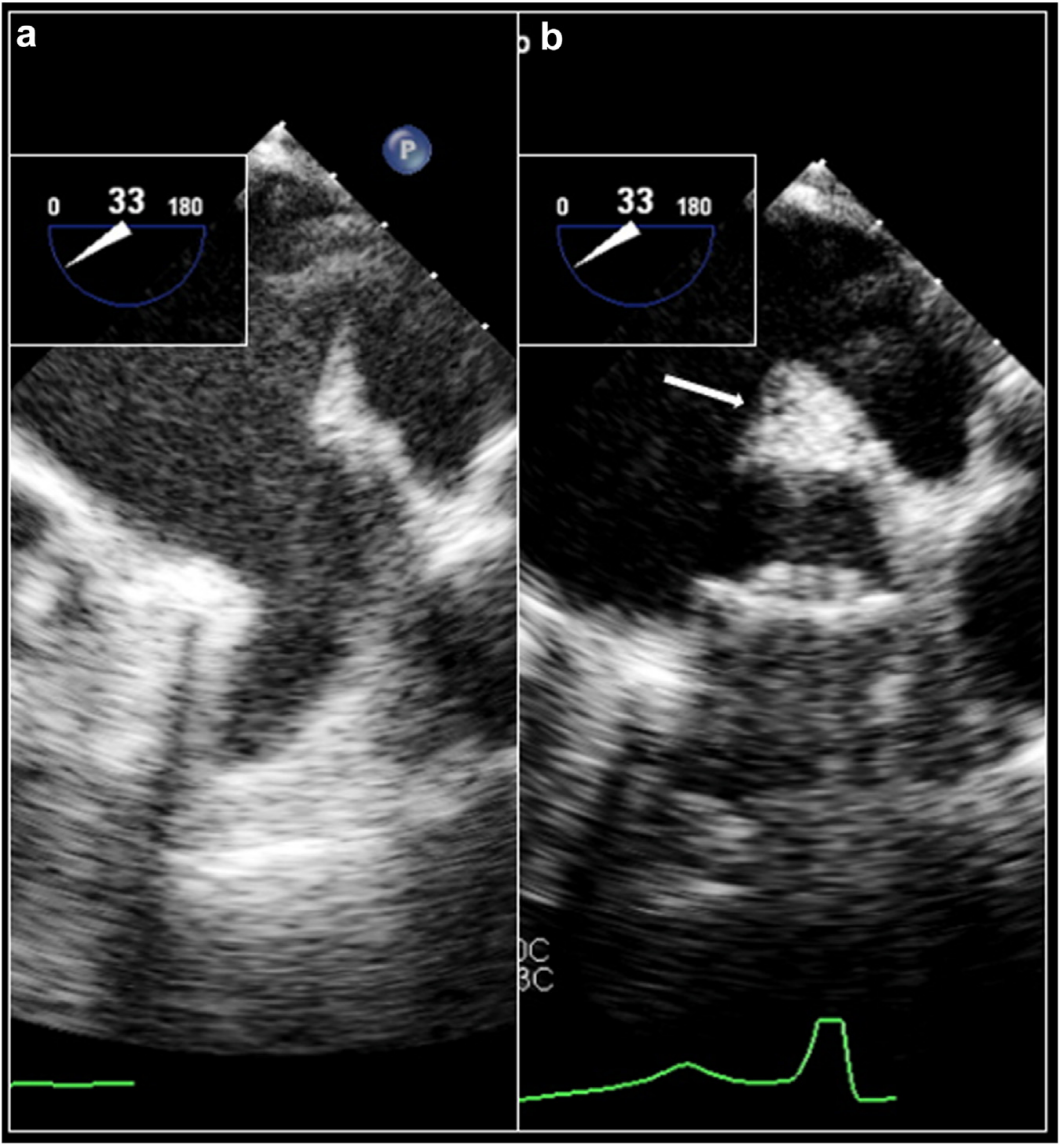
Coumadin Ridge Edema after AF ablation. Panel A: baseline images of the LAA prior to AF ablation. Panel B: significant coumadin ridge edema post AF ablation. Device implanted slightly deeper within the LAA to avoid anchoring along the edematous ridge Abbreviations: AF, atrial fibrillation; LAA, left atrial appendage.

In patients undergoing combined LAAO and AF ablation, implanters should maintain close attention to anatomic changes to the LAA ostium pre- and post-ablation particularly with radiofrequency ablation (RFA). In LAA anatomies with short coumadin ridges in relation to the LAA ostium, or where extensive RFA was applied to the base of the appendage, careful inspection of anatomy pre- and post-ablation and its relationship to the device landing zone is important. In a study of 98 patient undergoing AF ablation with concomitant LAAO vs. LAAO alone, there was a significant difference in new PDL rate among the two groups at 45 days (25.5% vs. 8.5%).^47^ Consideration of pre- and post-ablation TEE or ICE image acquisition to delineate these findings is imperative. Practically, when extensive edema along the coumadin ridge or base of the appendage is present after ablation, and device stability is dependent on anchoring along those regions, it may still be beneficial to consider staging LAAO once edema along the coumadin ridge has subsided (Figure 8). This will likely be less relevant in the future as many ablations transition to being done using pulse field ablation.

Depending on LAA anatomy and orientation of the working depth as well as the modality of catheter ablation used, trans-septal puncture at times does need to be repeated to allow for successful LAAO. In patients undergoing catheter ablation with RFA, a transseptal site accommodating both procedures, AF ablation and LAAO, is often achievable; however, this becomes more challenging in patients undergoing catheter ablation with “single-shot” solutions such as cryoablation or pulse field ablation, where trans-septal sites are usually anterior in the fossa.

## Role of Intracardiac Echocardiography (ICE) in LAAO

Initial experience as well as prevailing current practice for imaging to guide successful LAAO has predominantly been through TEE imaging owing to its excellent resolution and advances in recent years allowing more extensive two-dimensional (2D) and three-dimensional (3D) image manipulation. Since the advent of percutaneous LAAO, single-case reports and small series have suggested the feasibility of procedural success using ICE guidance.^48^^,^^49^ Several theoretic benefits have motivated the push toward using ICE more frequently including: obviating the need for general anesthesia, shorter length or stay and hospital resource utilization, and reduction in risk of complications related to TEE.

In 2023, the ICE-LAA study reported results examining the feasibility of LAAO using ICE guidance among 100 patients who underwent LAAO in a prospective single arm using a traditional 2D ICE catheter.^50^ Complete LAA seal was achieved in 98.5% of patients with 1.5% demonstrating 3 to 5 mm leaks at implant. On follow-up at 45 days, 74.7% of patients had ongoing total seal with 22.7% demonstrating leaks < 3 mm in size in keeping with previously published leak rates. Eighty-five percent of patients underwent imaging follow-up in the study (76% of all patients underwent follow up with TEE). While this study demonstrated acceptable success rates at implant and 45 days, the leak rate during follow-up perhaps could be an artifact of incomplete imaging at implant owing to the use of 2D ICE catheters.

With the advent of newer generations of 3D/4D ICE catheters, these limitations may become less pertinent. Several catheter solutions are commercially available, all of which house a larger number of ultrasound crystals at the catheter tip allowing for higher-resolution images as well as the ability of manipulate images in 2D and 3D without the need to reposition the ICE catheter as extensively.^51^

### Practical Considerations for 3/4D-ICE-Guided Implants

#### Preprocedure

Planning for an ICE-guided implant starts with baseline imaging of the LAA using a CT or reviewing baseline TEE images, if available. Preprocedure imaging is important to understand the LAA anatomy prior to vascular access and its amenability for closure and also to obtain adjunctive information on device sizing, trans-septal site, and delivery catheter selection. These data points are now increasingly made available using dedicated image processing suites that simulate device implants.^52^^,^^53^

#### Intraprocedure

**Figure 9:**
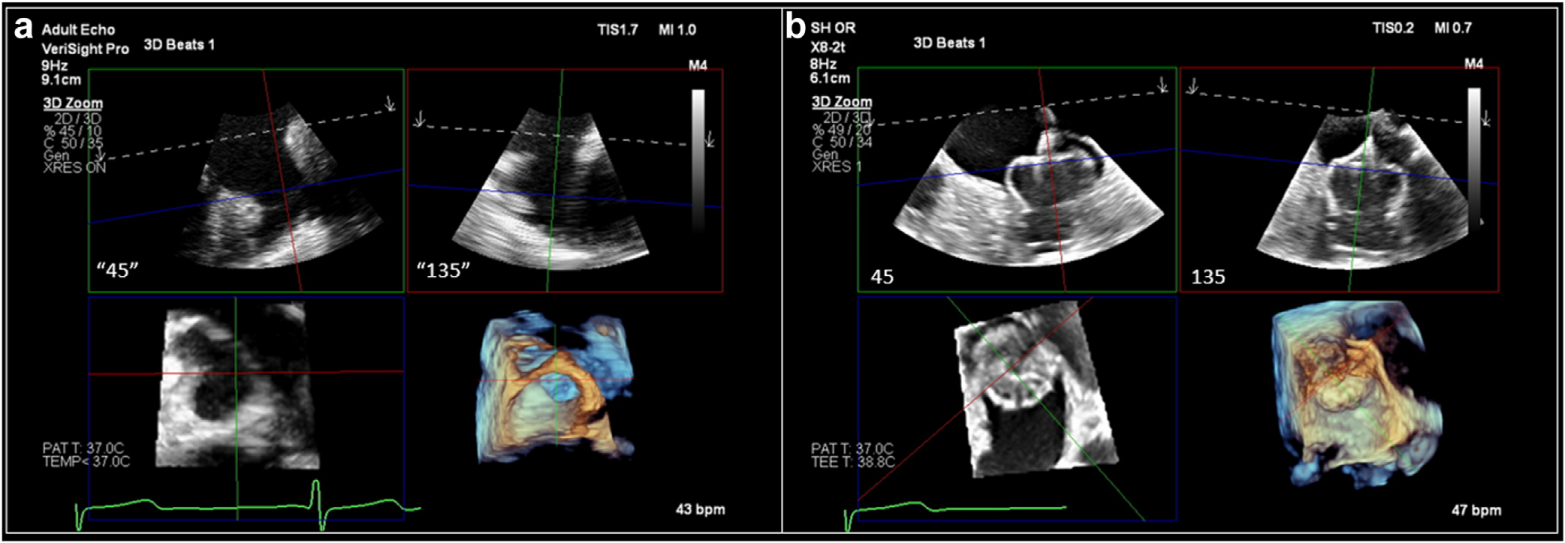
ICE image analysis using 3D MPR to mimic traditional TEE views. Panel A: baseline LAA images from the mid-LA vantage point manipulated to mirror traditional TEE views. Panel B: postimplant TEE images demonstrating identical anatomy to baseline ICE images with a well-seated Watchman device in place Abbreviations: 3D, three-dimensional; ICE, intracardiac echocardiography; LA, left atrial; LAA, left atrial appendage; MPR, multiplanar reconstruction; TEE, transesophageal echocardiography.

**Figure 10:**
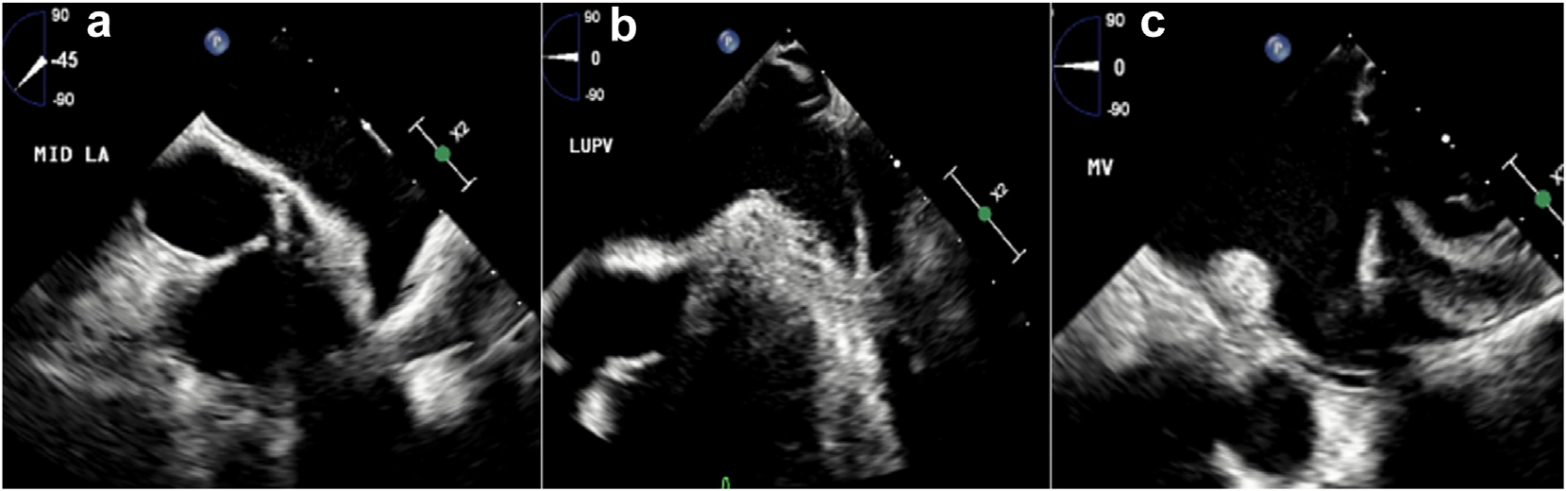
Key ICE imaging views of the LAA using 2D imaging. Panel A: mid-left atrial (LA) view. Panel B: left upper pulmonary vein (LUPV) view. Panel C: mitral valve (MV) or transmitral view Abbreviations: 2D, two-dimensional; ICE, intracardiac echocardiography; LAA, left atrial appendage.

**Figure 11:**
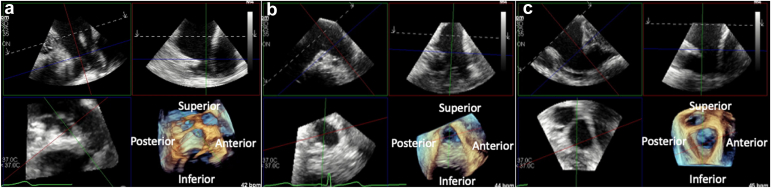
ICE 3D multiplanar reconstruction (MPR) of the LAA. Each panel corresponds to the similar panel from Figure 10. Labels are provided for spatial orientation. Panel A: mid-left atrial (LA) view. Panel B: left upper pulmonary vein (LUPV) view. Panel C: mitral valve (MV) or transmitral view Abbreviations: 3D, three-dimensional; ICE, intracardiac echocardiography; LAA, left atrial appendage.

The best vantage points to examine the LAA using ICE are all with the catheter tip inside the LA (adequate imaging may also be obtained in some patients with the catheter tip positioned in the pulmonary artery) (Figure 9). Three main catheter positions have been used as LA vantage points of the LAA: the mid-LA view, the trans-mitral view, and the left superior pulmonary vein view^54^ (Figure 10). Obtaining an optimal baseline 2D image is important to retain image resolution after manipulation. 3D multiplanar reconstruction can be valuable and is available using 3D ICE, similar to TEE (Figure 11).

In efforts to maintain effective communication between implanters and imagers as well as appropriate anticipatory delivery catheter movements, we suggest ICE image manipulation (whether in 2D biplane or 3D multiplane reconstruction) to mimic traditional TEE views as best possible (Figure 9). This allows predictability in appreciating anterior/posterior and inferior/superior anatomical relationships (Figures 10 and 11). After device implant, compression and color flow assessment should be done in 2D biplane imaging owing to the inferior resolution of 3D color reconstructed images. The ability to process 2D images with 3D ICE catheters in a stable position obviates the need to assess for color flow and compression from multiple different ICE catheter vantage points in most circumstances.

#### Limitations

Advances with 4D ICE catheter image acquisition and real-time processing present a substantial improvement over 2D ICE. Certain considerations continue to prevent ICE imaging from being the mainstream implant modality. First, TEE image quality particularly when using 3D multiplanar reconstruction, remains superior to ICE and inexperienced users may still prefer to use TEE guidance for this reason. Second, despite obviating the need for anesthesia/imaging personnel and potentially minimizing hospital length of stay, the inability to frequently reprocess the 4D ICE catheters in the same fashion as TEE probes and even 2D ICE catheters may present substantial long-term costs despite suggestion of comparable overall cost per patient encounter.^55^ Further, while preliminary data from smaller retrospective studies and larger registries suggest similar acute and 45-day success rates using ICE as compared to TEE, this has not been evaluated in a large prospective head-to-head study. Finally, safety concerns related to ICE catheter manipulation in the left atrium should be considered.

In a study by Ferro et al. comparing outcomes after ICE and TEE guided LAAO from the SURPASS nationwide LAAO registry, there was evidence of a higher rate of pericardial effusion in the ICE group compared to TEE at 45 days (1.0% vs. 0.5%; *p* ​= ​0.02). This was associated with lower operator experience with ICE-guided implants and seemed to ameliorate with increased operator experience over time, providing an opportunity for industry and implanters to collaborate on applying certain competency benchmarks and standardized procedural workflows to optimize results.^56^ In this regard, an Society for Cardiovascular Angiography and Intervention/Heart Rhythm Society expert panel recently advocated for the use of ICE-guidance of LAAO only among centers with experienced programs/implanters/imagers. While certainly not a limitation, it should be emphasized that especially when using the newer generation of 3D/4D ICE catheters (discussed below), the presence of a cardiac imaging specialist to provide appropriate image processing (i.e., using the 3D multiplanar reconstruction format) is also imperative.

## Funding

The authors have no funding to report.

## Disclosure Statement

G. W. Reed reports a relationship with Boston Scientific Corporation, Edwards Lifesciences, and Philips Healthcare that includes consulting or advisory services. The other authors had no conflicts to declare.
